# Denitrification Performance in Packed-Bed Reactors Using Novel Carbon-Sulfur-Based Composite Filters for Treatment of Synthetic Wastewater and Anaerobic Ammonia Oxidation Effluent

**DOI:** 10.3389/fmicb.2022.934441

**Published:** 2022-07-07

**Authors:** Yao Wang, Baorui Liang, Fei Kang, Youzhao Wang, Zhihong Yuan, Zhenning Lyu, Tong Zhu, Zhijun Zhang

**Affiliations:** ^1^Institute of Process Equipment and Environmental Engineering, School of Mechanical Engineering and Automation, Northeastern University, Shenyang, China; ^2^Shenyang Zhenxing Environmental Technology Co., Ltd., Shenyang, China

**Keywords:** anaerobic ammonium oxidation, autotrophs, carbon-sulfur-based composite filter, heterotrophs, shell powder

## Abstract

To avoid nitrate pollution in water bodies, two low-cost and abundant natural organic carbon sources were added to make up the solid-phase denitrification filters. This study compared four novel solid-phase carbon-sulfur-based composite filters, and their denitrification abilities were investigated in laboratory-scale bioreactors. The filter F_4_ (mixture of elemental sulfur powder, shell powder, and peanut hull powder with a mass ratio of 6:2.5:1.5) achieved the highest denitrification ability, with an optimal nitrate removal rate (NRR) of 723 ± 14.2 mg NO_3_^–^-N⋅L^–1^⋅d^–1^ when the hydraulic retention time (HRT) was 1 h. The HRT considerably impacted effluent quality after coupling of anaerobic ammonium oxidation (ANAMMOX) and solid-phase-based mixotrophic denitrification process (SMDP). The concentration of suspended solids (SS) of the ANAMMOX effluent may affect the performance of the coupled system. Autotrophs and heterotrophs were abundant and co-existed in all reactors; over time, the abundance of heterotrophs decreased while that of autotrophs increased. Overall, the SMDP process showed good denitrification performance and reduced the sulfate productivity in effluent compared to the sulfur-based autotrophic denitrification (SAD) process.

## Graphical Abstract

Novel filters for denitrification. 
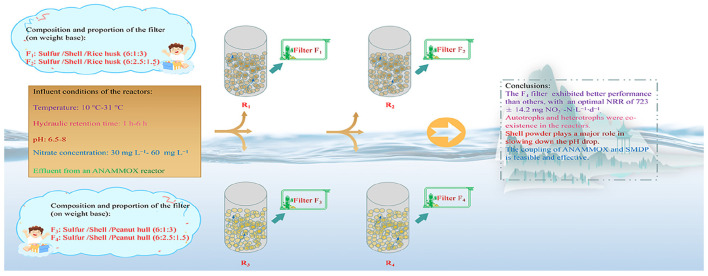

## Introduction

Nitrate, as a global contaminant, is frequently found in shallow groundwater, with adverse impacts on human health (e.g., methemoglobinemia and malformation) (Liang et al., 2021a). In addition, in saliva, the nitrites converted from nitrate might develop nitrosamines, which are known carcinogens, thus posing a huge risk to human health (Wang and Chu, 2016). Hence, removing nitrate in contaminated water is an urgent issue for the ecological environment.

Various methods have been proposed to remove nitrate, including electrodialysis, ion exchange, distillation, and biological processes. Compared to physico/chemical processes, biological denitrification is a cost-effective method which is widely used in wastewater treatment plants (WWTPs) to treat nitrate-polluted wastewater, and heterotrophic denitrification (HD) is the preferred biological method due to its favorable denitrification rate (Liang et al., 2021a; Vo et al., 2021). Nevertheless, water-soluble organic carbon is generally required for denitrification due to the low carbon/nitrate (C/N) ratio of nitrate-polluted wastewater, which complicates this process and increases the operation costs.


(1) $$55\,S_{0}+50\,{\mathrm{NO}}_{3}^{-}+38\,H_{2}\,\text{O}+20\,{\mathrm{CO}}_{2}+4\,{\mathrm{NH}}_{4}^{+}$$

$$\to 4\,C_{5}\,{\text{H}}_{7}\,{\text{O}}_{2}\,\text{N}+55\,{\mathrm{SO}}_{4}^{2-}+25\,N_{2}+64\,H^{+}$$

As a biological-based denitrification method, autotrophic denitrification can be used as an alternative way. Sulfur-based autotrophic denitrification (SAD) has received considerable attention in recent years (Sahinkaya et al., 2011; Chung et al., 2014; Liang et al., 2020). The use of elemental sulfur in a packed-bed reactor is a preferred method and has been applied in pilot and full-scale reactors (Sahinkaya et al., 2014). However, the solubility of elemental sulfur limits the denitrification rate, and the increased sulfate production may also be a problem (Eq. 1). Accordingly, a variety of factors were investigated for packed-bed reactor, including hydraulic retention time (HRT), temperature, nitrate loading rate (NLR), and the types of solid-phase filters (Sahinkaya et al., 2014; Vo et al., 2021). Regarding the latter, previous research used a mixture of elemental sulfur and inorganic carbon particles, while this is not as effective as the denitrification filter formed by the thermal fusion of elemental sulfur and inorganic carbon sources (Liang et al., 2020). Moreover, solid organic carbon sources have also been explored [polycaprolactone (PCL), starch/PCL, etc.], and their denitrification levels for wastewater were moderate (Chu and Wang, 2011).

To overcome these problems, considering that mixotrophic denitrification (simultaneous autotrophic and heterotrophic) could compensate for the disadvantages of autotrophic and heterotrophic denitrification (Sahinkaya and Dursun, 2012), and because most of the liquid carbon sources are costly, the solid-phase-based mixotrophic denitrification process (SMDP) was investigated. So far, only a limited number of studies have focused on the SMDP: For example, the effects of different pH values on SMDP and the selection of suitable filters have not been systematically studied. Moreover, as an efficient and economical process for wastewater treatment, anaerobic ammonium oxidation (ANAMMOX) has been successfully applied and found to reduce the amount of aeration required for nitrogen removal (Gilbert et al., 2014). It is a chemolithotrophic process without organic carbon demand. The ANAMMOX process is the anoxic oxidation of ammonium with ammonium acting as the electron donor to produce nitrogen gas (Eq. 2; Strous et al., 1998). However, the by-product nitrate would accumulate and further treatment was required, while there is very little information available regarding the performance of the SMDP coupled with the ANAMMOX, and the adoption of ANAMMOX effluent in SMDP may achieve desirable nitrogen removal performance.


(2) $${\text{NH}}_{4}^{+}+1.32\,{\text{NO}}_{2}^{-}+0.066\,{\text{HCO}}_{3}^{-}+0.13\,{\text{H}}^{+}\to 1.02\,{\text{N}}_{2}$$

+0.26⁢NO3-+0.066⁢CH⁢O0.52⁢N0.15+2.03⁢H2⁢O

In this study, two low-cost and abundant natural organic carbon sources were selected as HD materials and integrated into the SAD process, and parallel experiments were conducted in four SMDP reactors. The overall aims were as follows: (1) to compare the feasibility of four types of solid-phase filters for the SMDP; (2) to study the effects of influent pH on the denitrification ability of the SMDP; (3) to explore the feasibility of coupling SMDP with ANAMMOX; (4) to identify the variations in the microbial communities involved in the SMDP. These findings would provide a scientific basis for the development of measures to further improve wastewater treatment.

## Materials and Methods

### The Solid-Phase Filters

Four types of solid-phase filters were prepared for the SMDP. The elemental sulfur powder mixed with shell powder and rice husk powder according to the weight ratios of 6:1:3 and 6:2.5:1.5 was used to prepare F_1_ and F_2_, respectively. Filters F_3_ and F_4_ were prepared by mixing elemental sulfur powder with shell powder and peanut hull powder at weight ratios of 6:1:3 and 6:2:1.5, respectively. The mixed powders were, respectively, stirred under 150°C to form the molten material, which was poured into oval-shaped molds and naturally cooled to form the F_1_–F_4_ filters. The final products were in oval form, with a width of 5 mm, a height of 6 mm, and a length of 10 mm on average. The shell powder, elemental sulfur powder, rice husk powder, and peanut hull powder were obtained from a local technology company in Shenyang (Dongyuan, China), with a mean diameter of 600, 1500, 300, and 300 mesh, respectively.

### Experimental Procedure

Four identical up-flow packed-bed column reactors (R_1_–R_4_) were conducted in this study. The reactors were made of plexiglass, with a height-to-diameter ratio of 6:1 and an effective volume of 1 L. The R_1_–R_4_ reactors were, respectively, filled with filters F_1_, F_2_, F_3_, and F_4_, with 1 kg per filter type. The R_1_–R_4_ reactors were conducted in parallel and operated continuously for 320 days, and the Tables 1, 2 list the detailed influent parameters.

**TABLE 1:** Influent conditions of the R_1_–R_4_ reactors in periods 1–6.

Periods	1	2	3	4	5	6
Days	0–5	6–20	21–40	41–60	61–80	81–180
HRT (h)	6	4	3	2	1	1
NLR (mg NO_3_^–^-N⋅L^–1^⋅d^–1^)	200	300	400	600	1200	720
NO_3_^–^-N (mg L^–1^)	50					30
Average temperature (°C)	12	13	18	19	23	26

**TABLE 2:** Influent conditions of the R_1_–R_4_ reactors in periods 7 and 8.

Period 7						Period 8		
	
Steps		1	2	3	4	1	2	3
Days	Batch 1	181–185	186–190	191–195	196–200	261–280	281–300	301–320
	Batch 2	201–205	206–210	211–215	216–220			
	Batch 3	221–225	226–230	231–235	236–240			
	Batch 4	241–245	246–250	251–255	256–260			
HRT (h)	Batch 1	3				4	3	2
	Batch 2							
	Batch 3	1						
	Batch 4							
NO_3_^–^-N (mg L^–1^)		60				62 ± 2.3	61 ± 3.2	61 ± 2.6
NO_2_^–^-N (mg L^–1^)						5.3 ± 1.3	5.8 ± 1.1	5.7 ± 1.4
NH_4_^+^-N (mg L^–1^)		2.2 ± 0.4				11.2 ± 3.3	11.9 ± 2.6	12.2 ± 2.4
Influent pH value		6.5	7	7.5	8	7.3–7.8		
Temperature (°C)		29 ± 2						

During periods 1–6, the reactors were operated at ambient temperatures to evaluate the impact of temperature changes on the SMDP; meanwhile, the HRT and the influent nitrate concentration were adjusted to change the influent NLR to estimate SMDP performance under different conditions. Experiments were carried out indoors, starting at the coldest time, with an average temperature of 12°C (period 1). Hydrochloric acid solution and sodium bicarbonate solution were used to adjust the pH of the influent in period 7, fed-batch experiments were carried out to study the effects of different pH values on the performance of SMDP, batches 1 and 3 were performed in duplicate (corresponding to batches 2 and 4, respectively), and the HRT was also varied in period 7 (Table 2). Tap water supplemented with NaNO_3_ agent was used as synthetic wastewater for the influent of the R_1_–R_4_ reactors.

During period 8, the influent of the R_1_–R_4_ reactors was changed to the effluent from a 6000 L ANAMMOX reactor in our laboratory (Supplementary Figure 1) and operated under various conditions, and the detailed influent parameters are summarized in Table 2. The effluent collected from the ANAMMOX reactor was reserved in a middle tank and then evenly pumped into the R_1_–R_4_ reactors. The influent temperature of the R_1_–R_4_ reactors was maintained at 29 ± 2°C during periods 7 and 8, and the dissolved oxygen (DO) was not controlled throughout the study. The seed sludge was taken from the bottom of the secondary sedimentation tank of a local WWTP (Shenyang, China), and the reactors were, respectively, inoculated with 100 mL seed sludge (with mixed liquor suspended solids (MLSS) of about 5.4 g L^–1^) and operated under internal recycle mode with a flow rate of 15 L h^–1^ for 2 h to ensure an even distribution of the sludge throughout the reactor. After the internal recycle mode, the reactors were continuously fed with influent. The HRT was calculated considering the empty bed volume, and the HRT, NLR, and nitrate removal rate (NRR) were, respectively, calculated by the following equations:


(3) $$H\,R\,T=\frac{R_{v}}{S_{p}}$$

(4) $$N\,L\,R=\frac{C_{a}}{H\,R\,T}\times 24$$

(5) $$N\,R\,R=\frac{\left(C_{\text{a}}-C_{\text{b}}\right)}{H\,R\,T}\times 24$$

where *R*_*V*_ is the empty bed volume of the reactor (L); *S*_*p*_ is the influent flow rate of the reactor (L h^–1^); *Ca* is the influent nitrate concentration of the reactor (mg L^–1^); and *C*_*b*_ is the effluent nitrate concentration of the reactor (mg L^–1^).

### Sampling and Analysis

The water samples were collected daily from the influent and effluent of the R_1_–R_4_ reactors, and the samples were filtered using 0.45-μm cellulose acetate membrane and analyzed for chemical oxygen demand (COD), MLSS, ammonium, nitrite, nitrate, suspended solids (SS), sulfate, and alkalinity according to standard methods (American Public Health Association [APHA], 2005). The pH and DO values were measured with digital instruments (VSTAR10, Thermo Fisher, China, and BDO-209F, Bell, China, respectively). For the sulfide measurements, the methylene blue method was adopted (Wang et al., 2016). The hemicellulose, lignin, and cellulose contents of rice husk powder and peanut hull powder were measured according to the methods of Hill et al. (1998).

### DNA Extraction and Illumina MiSeq Sequencing

To identify the richness and diversity of microbial communities, the bio-samples on the surface of filters F_1_, F_2_, F_3_, and F_4_ in the R_1_–R_4_ reactors were collected. One bio-sample was collected from the top area of the R_1_–R_4_ reactors on days 80 and 320, respectively. The DNA was extracted using a PowerSoil DNA extraction kit (MP Biomedicals, United States), and after the sample collection, the extracted DNA was immediately placed at −20°C and then stored at −80°C until the next step. The V3–V4 hypervariable regions of 16S rRNA genes were amplified using the primers 338F (5′-ACTCCTACGGGAGGCAGCAG-3′) and 806R (5′-GGACTACHVGGGTWTCTAAT-3′). The AxyPrep DNA Purification Kit (AXYGEN, United States) was used to purify the PCR products. Afterward, all the purified 16S amplicons were pooled in equimolar and paired-end sequenced (2 × 300) on an Illumina MiSeq platform (Illumina, San Diego, CA, United States) in a biomedical laboratory (Majorbio, Shanghai, China). The raw 16S rRNA sequences have been deposited in the NCBI Sequence Read Archive (SRA) database under accession numbers PRJNA835999 and PRJNA836004.

## Results and Discussion

### Performance of the Bioreactors

**FIGURE 1:**
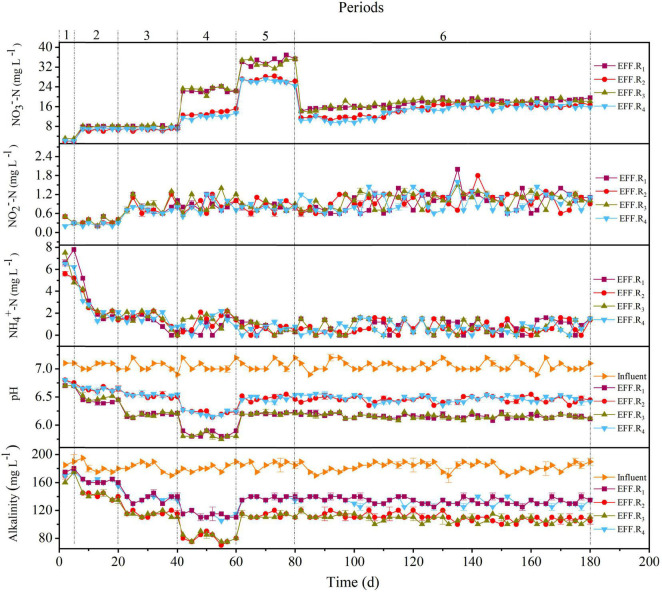
Performances of the R_1_–R_4_ reactors during periods 1–6.

**FIGURE 2:**
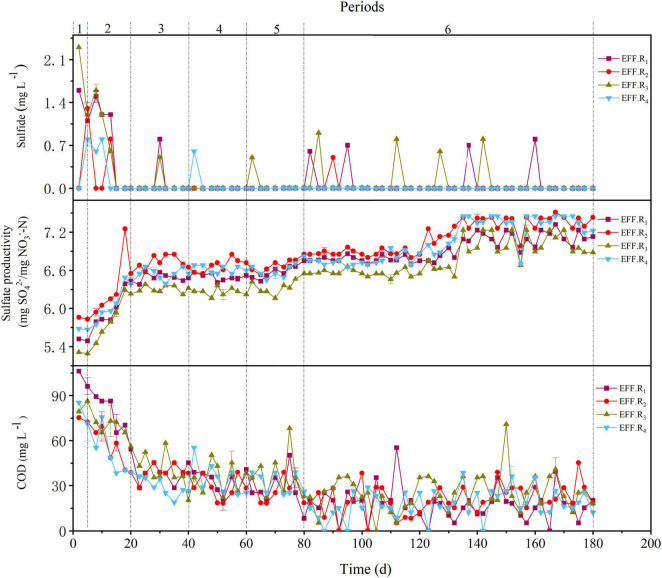
Variations in sulfide, COD, and sulfate productivity in periods 1–6 of the R_1_–R_4_ reactors.

The performances of the R_1_–R_4_ reactors are depicted in Figures 1, 2. The nitrate was almost entirely denitrified within 3 days in all reactors, indicating that the F_1_–F_4_ filters could start-up rapidly even at low temperatures (11–13°C) and display good denitrification performance. During period 1, a slight accumulation of ammonium was observed in R_1_–R_4_ reactors (Figure 1), which may be attributed to the dissimilatory nitrate reduction to ammonium (DNRA), caused by the high carbon-to-nitrogen ratio (C/N) (Rijn et al., 2006). To avoid such unfavorable factor, the HRT of the reactors was reduced in the period 2. However, all reactors showed a decrease in nitrate removal efficiency (NRE), which was 84.5 ± 4.1%, 86.9 ± 3.3%, 83.9 ± 2.6%, and 86.1 ± 1.8% for R_1_, R_2_, R_3_, and R_4_, respectively. Interestingly, the NRE of the reactors in the period 3 was similar as the period 2, even though the HRT in the period 3 was 3 h. The reason for this phenomenon may be due to the temperature fluctuations, as a lower temperature (≤15°C) could negatively affect the denitrification phenomenon (Sahinkaya et al., 2014), and compared with the period 2 (13°C), a higher temperature was observed in the period 3 (18°C). The HRT was further decreased in periods 4–5, the decreased NRE and the elevated nitrate concentrations in effluent were observed in all reactors, and the solubility of the solid-phase filter could be the main reason for the incomplete denitrification (Koenig and Liu, 2001). Above all, these results clearly indicated that the NRR was dependent of the HRT and temperature variation, and when the HRT was 2 h or 1 h in this study, it was hard to keep the effluent nitrate concentration always below 15 mg L^–1^, indicating that it was difficult for F_1_–F_4_ filters to take into an account both a relatively faster flow rate (HRT ≤ 2 h) and a high influent nitrate concentration (50 mg L^–1^). Given this scenario, the influent nitrate concentration was decreased to 30 mg L^–1^ to test the overall performance of the reactors in period 6.

In the first 15 days of period 6, the NRE of the R_1_, R_2_, R_3_, and R_4_ reactors was 53.5 ± 3.1%, 66.4 ± 3.6%, 58.9 ± 2.6%, and 70.1 ± 1.9%, respectively; and the NRE of the remaining days of period 6 (days 96–180) in the reactors was 39.5 ± 3.5%, 46.4 ± 2.7%, 41.9 ± 2.6%, and 50.1 ± 1.9%, respectively. This was in accordance with a previous study in packed-bed denitrification systems (Liang et al., 2021b). Although the average temperature in the reactors was 31°C during the last few days of period 6, which was closer to the optimum temperature of 35°C for nitrate reduction (Chen et al., 2018) than that in the other periods, the NRE values were still moderate. This may be due to the fact that after the filter had been working for a period of time, the release capacity of the organic carbon sources in the filter was weakened, which affects the denitrification capacity of the filter, because the HD rate was higher than the autotrophic denitrification rate (Oh et al., 2001). During periods 3–6, the F_4_ filter showed a relatively higher denitrification performance than the others. Compared to the R_3_ reactor, the nitrate concentration was higher in the R_1_ effluent. Similarly, the nitrate concentration of the R_2_ effluent was higher than that of R_4_, suggesting that peanut hull may be more suitable as a denitrification source than rice husk. The cellulose, hemicellulose, and lignin contents of rice husk were 38.4, 20.3, and 21.6%, respectively, and 34.6, 18.2, and 28.2% for the peanut hull. Cellulose and hemicellulose are reported to be the carbon sources for HD process (Wen et al., 2010), but their contents in peanut hull are lower than those in rice husk, and the better denitrification performance obtained with peanut hull may be due to the structural advantage given by the combination of peanut hull and elemental sulfur and the detailed release capacity of the carbon sources in the filters. Moreover, during periods 5–6, it was observed that the denitrification rates of the F_1_ and F_3_ filters were lower than those of F_2_ and F_4_, indicating that the content of natural organic carbon sources was not always proportional to the NRR, and a reasonable content of natural organic carbon sources may contribute to better denitrification. Notably, an optimal NRR of 723 ± 14.2 mg NO_3_^–^-N⋅L^–1^⋅d^–1^ was observed in the R_4_ reactor (period 5), which demonstrated superior denitrification capacity compared to the other packed reactors (Table 3).


(6) $$4\,{\text{S}}^{0}+4\,{\text{H}}_{2}\,0\to 3\,{\text{H}}_{2}\,\text{S}+{\text{SO}}_{4}^{2-}+2\,{\text{H}}^{+}$$

**TABLE 3:** Comparison of studies on packed-bed denitrification.

Type of the reactor	Type of the packed filter	HRT (h)	Temperature (°C)	The maximum denitrification rate (mg-N⋅L^–1^⋅d^–1^)	References
Column reactor	PHBV-Sawdust	1.5	/	146	Yang et al., 2020
Column reactor	Sulfur/Limestone	24–3	6–28	300	Sahinkaya et al., 2014
Column reactor	PCL/Starch	2–0.5	15–25	640	Shen et al., 2013
Column reactor	Polybutylene succinate/Bamboo powder	4	26	340	Qi et al., 2020
Column reactor	Sulfur/Shell/Peanut hull	6–1	10–31	723	This study

As shown in Figure 2, accumulations of COD were observed in all reactors during periods 1–2, with the average COD concentration of R_1_, R_2_, R_3_, and R_4_ reactors was 81.8 ± 31.2 mg L^–1^, 58.6 ± 28.1 mg L^–1^, 71.1 ± 35.8 mg L^–1^, and 56.6 ± 28.6 mg L^–1^, respectively. With the improvement of NLR in later periods, the average effluent COD concentration in all reactors decreased and reached an acceptable value (<50 mg L^–1^). During periods 5–6, the average effluent COD concentration of all the reactors was similar, whereas the average NRR in R_1_ and R_3_ was lower than that in R_2_ and R_4_. The respective sulfate productivity values in the R_1_, R_2_, R_3_, and R_4_ reactors were 6.68 ± 0.33 mg SO_4_^2–^/mg NO_3_^–^-N, 6.89 ± 0.29 mg SO_4_^2–^/mg NO_3_^–^-N, 6.51 ± 0.32 mg SO_4_^2–^/mg NO_3_^–^-N, and 6.78 ± 0.34 mg SO_4_^2–^/mg NO_3_^–^-N in periods 1–6, which were lower than the theoretical value (7.54 mg SO_4_^2–^/mg NO_3_^–^-N) calculated by Eq. 1. The accumulation of sulfate indicates that denitrification may still be dominated by SAD, and the lower sulfate productivity may be attributed to HD; specifically, the lower sulfate productivity of R_1_ and R_3_ may be attributed to the higher proportion of HD in the reactors. Moreover, the lower NRR in R_1_ and R_3_ may be related to the lower pH and alkalinity rates, and the effluent pH values in R_2_ and R_4_ were generally around 6.5 in periods 5–6, whereas in R_1_ and R_3_, these pH values were about 6.0–6.2. Generally, the pH range suitable for denitrification is 6.5–8.5 (Wang and Chu, 2016), the lower NRE of R_1_ and R_3_ may be due to the low content of the shell powder contained in the filters, because the content of shell powder in filters F_2_ and F_4_ was 2.5 times that of F_1_ and F_3_, and shell powder has a good effect on maintaining good denitrification performance due to its favorable alkalinity dissolution rate (Moon et al., 2004). Sulfide was occasionally found in the reactors and generally appeared in the R_1_ and R_3_ (Figure 2). The presence of sulfide might be attributed to the sulfur disproportionation process (Eq. 6), which is induced by the low nitrate load of the influent (Liang et al., 2021b). The higher sulfide content in R_1_ and R_3_ reactors in period 6 may be caused by the uneven distribution of denitrification capacity in the reactors. In general, the various proportions and compositions of the filter strongly affected the denitrification performance, and the F_4_ could be the optimal filter due to its overall performance. Besides, the F_4_ filter was composed of several low-cost substances and showed an excellent denitrification performance under different conditions, which further proves its economy and feasibility.

### Effect of pH on the Solid-Phase-Based Mixotrophic Denitrification Process

**FIGURE 3:**
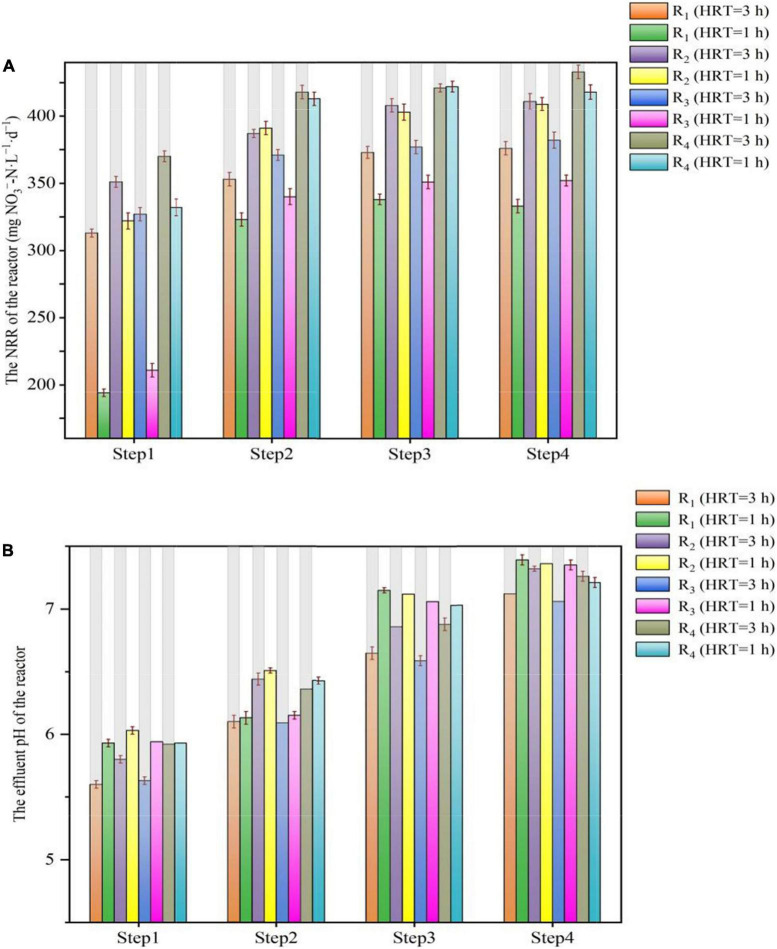
Variations in NRR and effluent pH value in period 7 of the R_1_–R_4_ reactors. **(A)** NRR; **(B)** effluent pH value.

The influent pH variations of the reactors under different conditions are depicted in Figure 3. When the HRT was 3 h, the NRR of the reactors was positively associated with an increase in pH value. During step 4, the average NRR of the R_1_, R_2_, R_3_, and R_4_ reactors was 376 ± 16.3 mg NO_3_^–^-N⋅L^–1^⋅d^–1^, 411 ± 20.1 mg NO_3_^–^-N⋅L^–1^⋅d^–1^, 382 ± 23.4 mg NO_3_^–^-N⋅L^–1^⋅d^–1^, and 433 ± 17.7 mg NO_3_^–^-N⋅L^–1^⋅d^–1^, respectively. These values were slightly higher than the NRR at pH of 7 or 7.5 in the four reactors (Figure 3A). On the contrary, when the pH was 6.5, the NRR of all the reactors showed a decreasing trend, and this may be due to the negative impact of the lower pH on denitrification (Figure 3B), because when the pH was lower than 6, denitrification will be inhibited (Yang et al., 2016).

When the HRT was 1 h, as the pH increased from 7.0 to 7.5, the NRR of the R_1_ and R_3_ reactors increased slightly, with the average NRR of the R_1_, R_2_, R_3_, and R_4_ reactors was 338 ± 12.8 mg NO_3_^–^-N⋅L^–1^⋅d^–1^, 403 ± 16.8 mg NO_3_^–^-N⋅L^–1^⋅d^–1^, 351 ± 19.6 mg NO_3_^–^-N⋅L^–1^⋅d^–1^, and 422 ± 15.2 mg NO_3_^–^-N⋅L^–1^⋅d^–1^, respectively. When the pH further increased to 8.0, the NRR of the four reactors hardly changed. Notably, when the pH decreased to 6.5, the NRR of all reactors began to decrease; in particular, the NRR in R_1_ and R_3_ decreased by about 40% compared to that at pH 7.5, whereas in R_2_ and R_4_, this value decreased by approximately 20%. These findings showed that the lower pH could significantly decrease the NRR, although the NRR also declined when the HRT was 3 h, and the decline range was not as large as that with HRT of 1 h. Meanwhile, the increased pH value did not bring better NRR to all the reactors, while only a limited increase in NRR of the R_1_ and R_3_ reactors was observed. The reason for these phenomena may be attributed to the solubility of the solid-phase filter, and it could be speculated that as the pH decreases, even if the HD caused by the natural solid organic carbon sources can counterbalance a certain drop in pH (Liang et al., 2021b), while shell powder would be the major role to slow down the pH drop. As the flow rate in the reactor increased, it was difficult for the solid-phase shell powder to provide sufficient alkalinity to alleviate the drop in pH, and the rapid drop in pH led to a decrease in NRR. This might explain why the NRR of the R_1_ and R_3_ was more susceptible to the decrease in influent pH value. Besides, when the HRT was 1 h, although a gradual increase in pH from 6.5 to 8.0 is theoretically beneficial for denitrification (Pang and Wang, 2021), there are limited electron donors released by elemental sulfur and natural organic carbon sources at this time. Hence, denitrification showed little benefit even if the pH increased.

Above all, the pH variations affected the NRR of the SMDP, and the rate of denitrification was affected by insufficient alkalinity supply when the influent pH was around 6.5. On the contrary, changes in pH will also affect the microbial activity and thus the denitrification capacity (Chen et al., 2018). When the electron donor in the reactor was insufficient, even if the pH was raised to the ideal range, it could hardly help the denitrification. Therefore, when SMDP or other denitrification reactors are adopted to remove the nitrate, the suitable pH should be considered in conjunction with the specific operation conditions (HRT, nitrate concentration, temperature, etc.).

### Performance of the Solid-Phase-Based Mixotrophic Denitrification Process Coupled With Anaerobic Ammonium Oxidation

**FIGURE 4:**
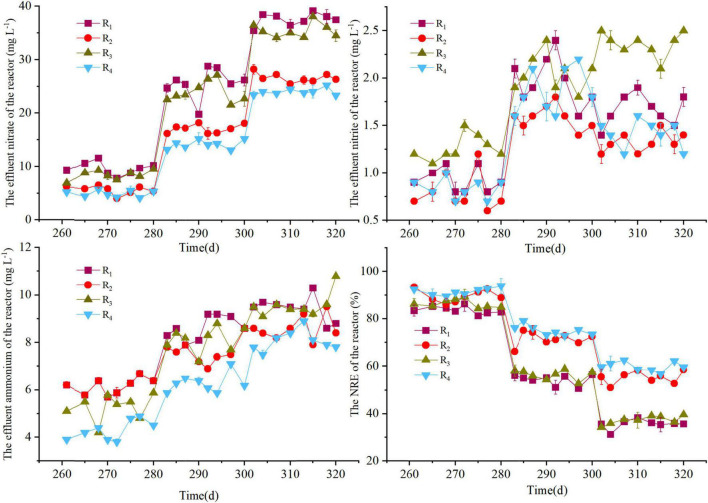
Variations in nitrate, nitrite, ammonium, and NRE in period 8 of the R_1_–R_4_ reactors.

As shown in Figure 4, when the HRT was 4 h (step 1), the low nitrogen levels were observed in the effluent of the R_2_ and R_4_ reactors, with the average ammonium concentrations were also controlled within 5 mg L^–1^, whereas the sum of the concentrations of nitrate, nitrite, and ammonium in the effluents of R_1_ and R_3_ reactors was greater than 15 mg L^–1^. Lowering the HRT to 3 h in step 2 resulted in the nitrate accumulation in the reactors, with the NRE of the R_1_, R_2_, R_3_, and R_4_ reactors was 53.7 ± 3.1%, 71.5 ± 2.7%, 56.9 ± 2.6%, and 76.8 ± 3.2%, respectively. Further reduction in HRT from 3 h to 2 h in step 3 resulted in increased nitrate levels in all reactors, and the average nitrate concentration of the effluent in the R_1_ and R_3_ reactors was 38.6 ± 2.3 mg NO_3_^–^-N⋅L^–1^ and 37.8 ± 1.9 mg NO_3_^–^-N⋅L^–1^, respectively. Compared with periods 3–4, a decrease in NRR was observed in steps 2 and 3 (within period 8), which could be explained by the following reasons: (1) The denitrification capacity may be weakened due to the consumption and loss of electron donors (elemental sulfur and natural organic carbon). (2) The mass transfer of the filter and nitrate was inhibited due to the increased sludge amount in the reactors (Zhou et al., 2011), because the sludge in the reactor was not artificially removed throughout the experiment. In addition, the ammonium concentrations in steps 2 and 3 were generally above 5 mg L^–1^, and the optimal performance for the coupled system (SMDP and ANAMMOX) was obtained in step 1, as the HRT of the SMDP was decreased to 3 h or lower, an additional method (e.g., effluent reflux or backwashing) may be required to remove excess nitrogen to improve the quality of the effluent (Zhang et al., 2019; Liang et al., 2022).

In summary, coupling ANAMMOX with the SMDP can effectively reduce the excessive pollutant concentrations in ANAMMOX effluent. The HRT is an important factor for SMDP, which affects the overall performance of the coupled system. However, a potential problem of the coupled system was speculated because the average SS concentration in the ANAMMOX effluent was 42 mg L^–1^, which was similar as a previous ANAMMOX reactor (Zhang et al., 2018). Compared with the tap water or the effluent of secondary sedimentation tank (SS < 10 mg L^–1^), the high SS concentration of the ANAMMOX effluent may cause the blockage of SMDP, thereby affecting the overall denitrification performance, and the air–water backwashing method could be an effective way to remove excess sludge, which has been verified in a previous study (Zhou et al., 2021). Further verification of the optimization strategy for the coupled system is necessary in future research. Overall, as demonstrated in this study, the F_4_ filter represents an effective and more favorable performance throughout the study, which provided a novel and feasible method for the SMDP process and solid-phase-based denitrification technology.

### Comparative Analysis of Microbial Community

**FIGURE 5:**
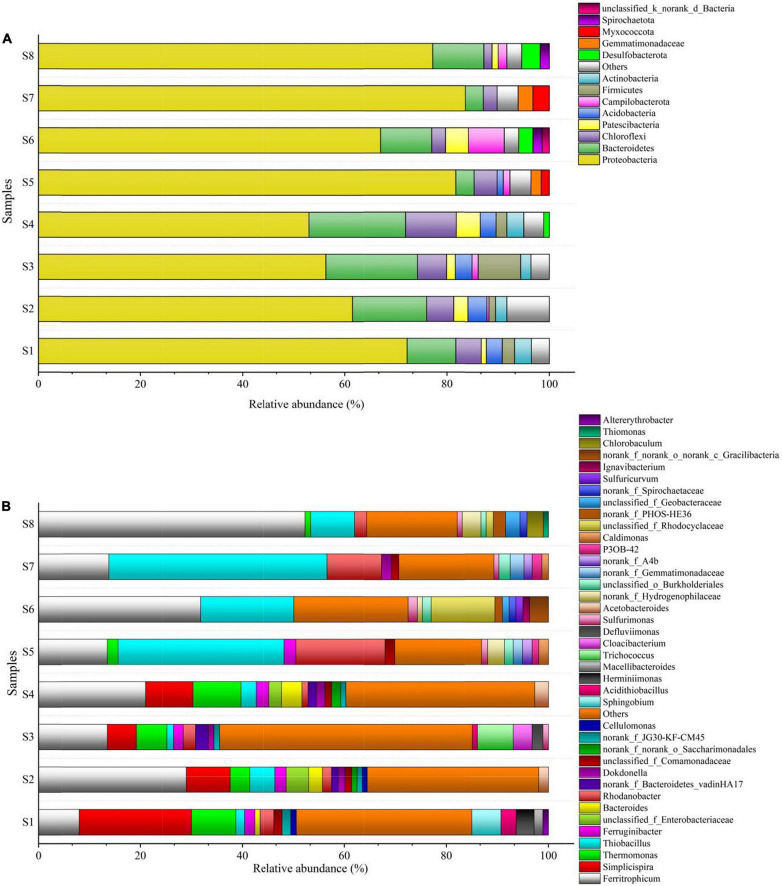
Taxonomic classification of the eight bio-samples at **(A)** phylum level; **(B)** genus level.

The results of the bacterial communities assigned to the phylum and genus levels, with the relative abundance of the most abundant (>1%), are summarized in Figure 5. Overall, 13 bacterial phyla were found in the eight bio-samples (Figure 5A). *Proteobacteria* were the largest phyla in all the bio-samples, which have been detected as the autotrophic denitrifying bacteria in previous study (Han et al., 2020). In all reactors, the relative abundance of *Proteobacteria* increased over time, from 53–72.2% at S_1_–S_4_ (S_1_, S_2_, S_3_, and S_4_: microbial communities on day 80 of the R_1_, R_2_, R_3_, and R_4_, respectively) to 67–83.6% at S_5_–S_8_ (S_5_, S_6_, S_7_, and S_8_: microbial communities on day 320 of the R_1_, R_2_, R_3_, and R_4_, respectively). The remaining dominant phyla were *Bacteroidetes* and *Chloroflexi*, and their abundances in S_5_–S_8_ were both decreased. *Bacteroidetes*, *Chloroflexi*, *Firmicutes*, and *Actinobacteria* are related to the biodegradation of organic matters (Miura et al., 2007; Xin and Qiu, 2021), and similar to the *Bacteroidetes* and *Chloroflexi*, the abundances of phyla *Firmicutes* and *Actinobacteria* also decreased over time. This shift might have been caused by changes in the amounts of organic carbon sources in the filters. Furthermore, increase in abundance of *Desulfobacterota* was observed in the R_2_ and R_4_, which is known as the sulfate-reducing bacteria (Frolov et al., 2021); meanwhile, the abundance of *Campilobacterota* in the R_2_ and R_4_ was also increased, *Campilobacterota* has been reported as the sulfide-oxidizing bacteria (Carrier et al., 2020), and the increase in its abundance was likely benefiting from the *Desulfobacterota* in the R_2_ and R_4_.

The genera *Ferritrophicum*, *Simplicispira*, and *Thermomonas* were found to be dominant in the bio-samples S_1_–S_4_ (Figure 5B). *Ferritrophicum* was reported as the denitrifying bacteria in the sulfur-based autotrophic system (Wan et al., 2019), and in this study, the abundance of *Ferritrophicum* increased in all the reactors. *Thiobacillus*, the typical denitrifying bacteria in SAD processes (Zhang et al., 2020; Dominika et al., 2021), was present at low abundances in S_1_–S_4_, and its abundance significantly increased in S_5_–S_8_. Conversely, the abundance of *Simplicispira* decreased to almost negligible levels in S_5_–S_8_, which probably due to its function was to degrade the organic carbon (Feng et al., 2017). The reason for these phenomena may be that the compositions of the filters changed with operation. The autotrophs and heterotrophs were both abundant in S_1_–S_4_. The heterotrophs in S_1_–S_4_, including *Ferruginibacter*, *Bacteroides*, *Trichococcus*, *Dokdonella*, *Herminiimonas*, *Sphingobium*, and *Cellulomonas*, are associated with the degradation of organics or HD processes (Chen et al., 2016; Han et al., 2020; Tian et al., 2021; Xin and Qiu, 2021; Zhang et al., 2021), and their abundances were both decreased or disappeared in S_5_–S_8_. The abundance of *Rhodanobacter* increased in R_1_ and R_3_ reactors, even though these bacteria are related to the degradation of carbohydrates (Xin and Qiu, 2021). *Ignavibacterium* has been reported as the nitrite-reducing bacteria (Chai et al., 2020) and was only found in S_7_, most likely because of the high nitrite concentration of the ANAMMOX effluent. *Chlorobaculum* is sulfur-oxidizing bacteria that could oxidize sulfide and elemental sulfur to sulfate (Zhang et al., 2021), its appearance in S_8_ may be attributed to the sulfur disproportionation process or the SAD process, whereas the sulfide was hardly found in the R_4_ effluent (Figure 2), and it is thus considered that the *Chlorobaculum* may give extra advantage to ensure the quality of the effluent.

In all reactors, the autotrophs and heterotrophs were both abundant on day 80; however, on day 320, the heterotrophs decreased significantly accompanied by the increase of autotrophs. Although the abundance of heterotrophs in S_5_ and S_7_ was higher than that in S_6_ and S_8_, the structure, proportion, and composition of the filter itself likely played a decisive role in the process of denitrification. Comparatively, the F_4_ filter showed a better performance under different conditions, providing a powerful option for optimizing the SMDP and the coupling of SMDP and ANAMMOX.

### Engineering Implications and Future Research

The SMDP-based denitrification system provided considerable nitrogen removal performance in laboratory-scale bioreactors, besides, the composition of the filters and the long-term experimental results also demonstrate its economy and reliability. Although the conventional HD processes are still widely applied in WWTPs, it is expected that the SMDP process can reduce the cost of denitrification, such as installing an SMDP device in the effluent of the secondary sedimentation tank, thereby reducing or avoiding the addition of organic carbon sources in the previous process (e.g., pre-denitrification tank). Similarly, compared to the HD processes, some scholars have found that the SAD processes were more cost-effective (Vandekerckhove et al., 2018; Wang et al., 2021). In addition, compared to the conventional nitrification/denitrification processes, the ANAMMOX process has the potential to save more than 90% of operational costs (Zhang et al., 2018), and it can be speculated that the sludge production would also be reduced through the ANAMMOX–SMDP coupled system. Furthermore, the implementation of SMDP process coupled with other efficient nitrogen removal processes [e.g., partial-denitrification– ANAMMOX (PD/A), partial-nitritation–ANAMMOX (PN/A), etc.] may be meaningful to improve nitrogen removal rates. In general, the SMDP process represents a good denitrification effect and has strong practical engineering significance.

## Conclusion

The solid-phase carbon-sulfur-based composite filters were successfully investigated in reactors. Changes in influent pH significantly impacted denitrification, and the content of shell powder played an important part in the filter to alleviate water acidification. The enhanced denitrification performance was observed in the R_4_ reactor, and the R_4_ was also the superior reactor in ANAMMOX–SMDP coupled systems. This study confirmed the overall performance of the SMDP, with F_4_ as a promising filter for the purification of nitrogen-contaminated wastewater (e.g., the effluent of the secondary sedimentation tank of WWTPs).

## Data Availability Statement

The data presented in this study are deposited in the NCBI SRA database under accession numbers PRJNA835999 and PRJNA836004.

## Author Contributions

YaW: conceptualization, data curation, investigation, formal analysis, writing – original draft, and visualization. BL: conceptualization, supervision, and editing. FK: conceptualization and editing. YoW: supervision and editing. ZY and ZL: supervision. TZ: funding acquisition, writing – original draft, and editing. ZZ: funding acquisition, supervision, writing – original draft, and editing. All authors contributed to the article and approved the submitted version.

## Conflict of Interest

ZY was employed by Shenyang Zhenxing Environmental Technology Co., Ltd. The remaining authors declare that the research was conducted in the absence of any commercial or financial relationships that could be construed as a potential conflict of interest.

## Publisher’s Note

All claims expressed in this article are solely those of the authors and do not necessarily represent those of their affiliated organizations, or those of the publisher, the editors and the reviewers. Any product that may be evaluated in this article, or claim that may be made by its manufacturer, is not guaranteed or endorsed by the publisher.
