# Supplementary material for: Denitrification Performance in Packed-Bed Reactors Using Novel Carbon-Sulfur-Based Composite Filters for Treatment of Synthetic Wastewater and Anaerobic Ammonia Oxidation Effluent
Source: Front Microbiol. 2022 Jul 7;13:934441. doi: 10.3389/fmicb.2022.934441 (PMC9301263; doi:10.3389/fmicb.2022.934441)
Supplement: Supplementary file 1 [file Data_Sheet_1.doc]

Supplementary Material


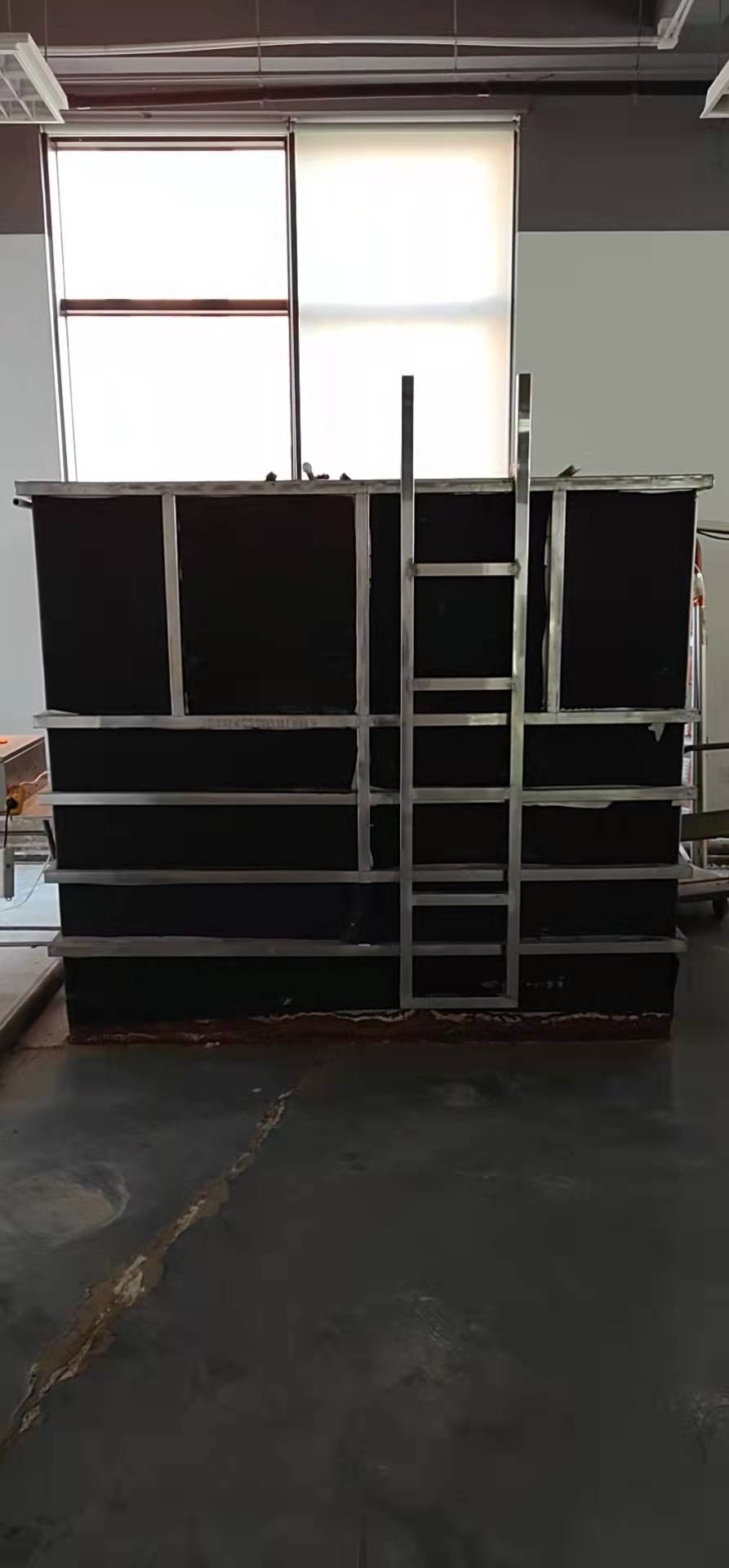


**FIGURE S1** **|** The ANAMMOX reactor in our lab.

The ANAMMOX reactor was made of stainless steel and in rectangular form with an effective volume of 6000 L. The water inlet of the reactor adopts the bottom-up mode, and several groups of membrane filters are placed inside. The reactor has been in continuous operation for more than two years with the HRT of 12 h, the temperature and pH of the reactor were maintained at 30 ± 1 °C and 7.5 ± 0.5, respectively. The synthetic wastewater used as the influent of the reactor that was composed of NH_4_Cl and NaNO_2_ as ammonium and nitrite source, respectively. The nitrogen loading rate (NLR) was maintained at 1.25 ± 0.1 kg-N m^−3^ d^−1^, and obtained an average nitrogen removal rate of 1.08 ± 0.03 kg-N m^−3^ d^−1^.
